# A Sedation Protocol for Endoscopic Baton‐Plate Adjustment in Neonates With Pierre Robin Sequence

**DOI:** 10.1002/pan.70286

**Published:** 2026-08-18

**Authors:** Nicolas Leister, Sarah Achterrath, Uwe Trieschmann, Teresa Kruse, Bert Braumann, Volker C. Schick

**Affiliations:** ^1^ Department of Anesthesiology and Intensive Care Medicine University of Cologne, Faculty of Medicine and University Hospital Cologne Cologne Germany; ^2^ Department of Orthodontics University of Cologne, Faculty of Medicine and University Hospital Cologne Cologne Germany

**Keywords:** difficult airway, pediatric anesthesia, pre‐epiglottic baton plate, procedural sedation, Robin sequence, standardized sedation protocol

## Abstract

**Background:**

Robin sequence (RS) is characterized by upper airway obstruction leading to life‐threatening desaturations in neonates. Procedural sedation in this population presents unique challenges due to anatomical airway anomalies, young patient age, and associated comorbidities. The pre‐epiglottic baton‐plate (PEBP) procedure is a non‐invasive, endoscopically guided intervention that alleviates obstruction, facilitates oral feeding, and promotes mandibular growth. Sedation for PEBP adjustment requires the maintenance of spontaneous respiration while ensuring patient comfort and procedural tolerance.

**Aims:**

To compare process characteristics and observed adverse events before and after an institutional change in the sedation regimen and staffing model for endoscopic PEBP adjustment in neonates with RS.

**Methods:**

This retrospective single‐center cohort study included 18 infants undergoing endoscopic PEBP adjustment between December 2012 and June 2025. Cohort I (*n* = 8) received individualized sedation, most commonly propofol with remifentanil, with variable personnel present. Cohort II (*n* = 10) received sevoflurane induction followed by ketamine and dexmedetomidine for maintenance, with predefined interdisciplinary staffing including two senior anesthesiologists. Demographic data, sedation regimens, sedation‐related adverse events, and airway interventions were compared between groups.

**Results:**

All 18 procedures were completed successfully. A total of five sedation‐related adverse events occurred, four in Cohort I and one in Cohort II. All events were managed effectively with routine measures. No patient required conversion to general anesthesia or invasive airway management. Procedural duration was significantly shorter in Cohort II. Baseline, lowest intraoperative, and first postoperative oxygen saturation did not differ significantly.

**Conclusions:**

Standardization of sedation protocols and procedural workflows was associated with shorter procedure times and fewer observed adverse events; however, the small sample size (*n* = 8 and *n* = 10) precludes conclusions regarding safety or causality. In rare, high‐risk neonatal procedures, structured and interprofessional approaches may help reduce variability and support reproducible care.

## Abstract (highlights)

**What Is Already Known About the Topic:**

Maintaining spontaneous breathing during procedural sedation for airway procedures in children with Robin sequence is challenging and requires a highly specialized medical team.

**What New Information This Study Adds:**

The introduction of standardized care bundles (standardized sedation protocol, structured team composition, defined procedural environment, perioperative monitoring strategy) accelerates the procedure, contributes to greater procedural consistency, and may reduce the incidence of adverse events, with a focus on patient safety.

## 1 Introduction

Robin Sequence (RS) is a rare congenital condition with an estimated incidence ranging from 1 in 8500 to 1 to 14 000 live births, depending on diagnostic criteria and the population studied [1, 2, 3]. RS is characterized by the triad of micrognathia, glossoptosis, and retroglossal airway obstruction [1, 4]. A cleft palate occurs in up to 90% of affected infants [5]. The etiology of RS is multifactorial, most commonly resulting from early developmental anomalies linked to genetic syndromes, oligohydramnios, facial myotonia or underlying connective tissue disorders [6, 7, 8]. The degree of upper airway obstruction varies widely. In severely affected neonates, posterior tongue displacement can lead to dynamic airway collapse, especially in the supine position, resulting in oxygen desaturation, feeding difficulties, and in severe cases, life‐threatening respiratory compromise [9].

Initial conservative therapy strategies include prone positioning, nasopharyngeal airways, palatal plates, and continuous or intermittent positive airway pressure. In severe or refractory cases, surgical interventions, such as tongue‐lip adhesion, mandibular traction, subperiosteal floor‐of‐mouth release, mandibular distraction osteogenesis, or tracheostomy may be required [4, 10].

In recent years, the pre‐epiglottic baton plate (PEBP) has gained recognition as a minimally invasive, endoscopically guided method to relieve airway obstruction. By anteriorly displacing the tongue base, the PEBP reduces glossoptosis‐related airway obstruction, improves mandibular‐lingual alignment, and mandibular growth, and facilitates functional jaw‐tongue coordination. For these reasons, PEBP is considered to be a causal treatment and may reduce the need for osteogenic distraction surgery [11]. These anatomical and functional improvements not only enable more effective swallowing and oral feeding, but also significantly reduce anesthesia risks [12, 13, 14].

Figure 1 illustrates the PEBP and the PEBP positioned for therapeutic use.

**FIGURE 1:**
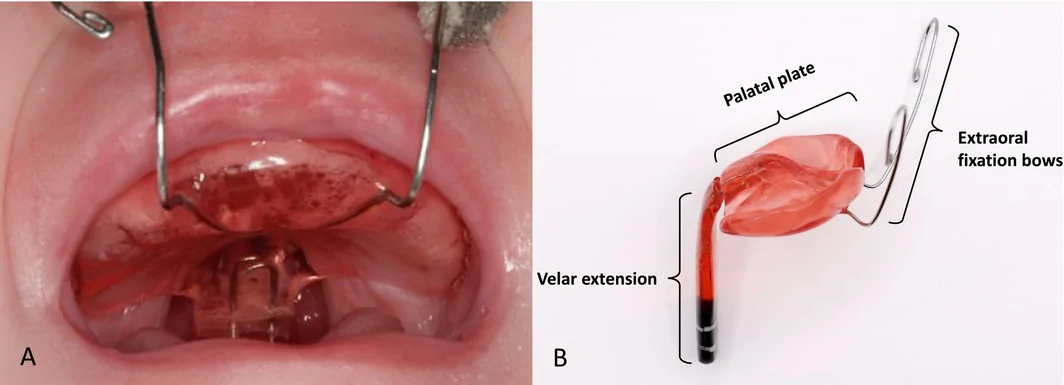
Pre‐epiglottic baton plate in therapeutic position. Figure 1 shows the pre‐epiglottic baton plate (B) and the pre‐epiglottic baton plate in situ (A), with its velar extension positioned posterior to the tongue base to reposition the tongue base forward and alleviate glossoptosis‐related upper airway obstruction.

For adjustment of the PEBP, children with a particularly high symptom burden undergo endoscopic fitting. At the authors' center, this procedure is performed under analgosedation in order to reduce procedural stress and risk; spontaneous breathing must be maintained.

However, airway management in RS remains highly challenging due to anatomical anomalies, dynamic airway obstruction, and the risk of rapid deterioration during sedation or anesthesia induction [15, 16]. Given the complexity of airway management in neonates with RS, this retrospective study aims to evaluate the feasibility, safety, and clinical impact of implementing a standardized care bundle for procedural sedation during endoscopic PEBP adjustment.

## 2 Methods

This retrospective, single‐center cohort study was conducted at the University Hospital of Cologne and approved by the institutional ethics committee (Chairperson Prof. Dr. R. Voltz; approval number 23‐1145_2, Amendment 08.09.2025). The study was carried out in accordance with the Declaration of Helsinki and current guidelines on patient safety in *Pediatric Anesthesia* [17].

### 2.1 Study Design and Patient Population

All neonates diagnosed with RS who underwent endoscopic adjustment of the PEBP under procedural sedation between December 2012 and June 2025 at the University Hospital of Cologne, Germany, were included. Only cases with clinically significant airway obstruction requiring PEBP adjustment were managed with endoscopic PEBP adjustment. Indications were based on documented signs of obstructive breathing (e.g., suprasternal retractions, noisy breathing, desaturation during sleep) and/or a mixed obstructive apnea–hypopnea index (MOAI) > 10 on polysomnography [18]. All patients required intensive perioperative monitoring and a multidisciplinary airway management approach.

Patients were divided into two cohorts based on the perioperative sedation approach:

- **Cohort I** (*n* = 8): Treated until 2022 using individualized sedation protocols.
- **Cohort II** (*n* = 10): Treated from 2022 onwards following a standardized sedation and procedural care bundle.

### 2.2 Standardized Care Bundle (Cohort II)

The standardized care bundle was implemented in 2022 after the participating disciplines had independently recognized that frequent changes in team composition were associated with considerable variability in experience in managing this clinical condition.

The standardized care bundle included the following key components:

- **Standardized sedation protocol**: Use of a predefined drug regimen (dosage and sequence) for analgosedation, designed to maintain spontaneous respiration while ensuring adequate procedural tolerance.
- **Structured team composition**: A dedicated interdisciplinary team comprising a senior pediatric anesthetist (supported by a consultant anesthetist), a pediatric orthodontist, an otolaryngologist, and experienced pediatric OR nurses. A pediatric pulmonologist is consulted as needed, based on the clinical indication.
- **Defined procedural environment**: All procedures in Cohort II were conducted in a pediatric operating theater, using a consistent spatial layout and equipment setup designed to optimize access to the airway and facilitate interdisciplinary coordination. In contrast to the variable settings in Cohort I, a fixed team structure and room setup were adhered to in each case, reflecting efforts to improve procedural safety through structural standardization.
- **Perioperative monitoring strategy**: All patients were admitted to the pediatric intensive care unit (PICU) for pre‐ and postoperative monitoring, including continuous cardiorespiratory surveillance and availability of immediate airway support.

### 2.3 Adjustment of the PEBP

Endoscopic adjustment of the PEBP represents a targeted approach to optimizing upper airway patency in infants with obstruction, particularly in the context of Pierre Robin sequence. For PEBP adjustment, flexible endoscopy is used to guide sequential modifications of the plate's angulation, length, and width in order to achieve sufficient anterior displacement of the tongue base and thereby alleviate airway obstruction. In the experience of the interdisciplinary team of this tertiary center, a certain degree of sedation is required for this procedure, as attempts at awake placement and adjustment have frequently been associated with procedural intolerance and compromise of the already vulnerable airway.

In recent years, an increasing proportion of neonates with RS at our institution have undergone endoscopic PEBP adjustment, reflecting a growing recognition of its clinical utility in managing upper airway obstruction. As procedural volume increased, it became evident that the previously individualized, case‐dependent approach was no longer adequate to meet the demands of consistent, high‐quality care. This prompted the development and implementation of a structured, standardized care pathway aimed at optimizing patient safety and procedural efficiency. The resulting best‐practice framework, ideally beginning with a reliable prenatal diagnosis, is grounded in interdisciplinary collaboration and integrates predefined protocols for sedation, team composition, procedural environment, and perioperative monitoring, each of which contributes to a more reproducible and qualitatively robust model of care [19].

### 2.4 Data Collection and Analysis

Patient records, anesthesia protocols, and perioperative documentation were retrospectively reviewed. We analyzed parameters including demographic data, sedation regimen, procedure duration, perioperative complications (e.g., desaturation, airway obstruction, unplanned intubation), and postoperative course (PICU stay, need for escalation of respiratory support). Adverse events and measures were documented according to established clinical standards. Data collection was performed by two independent reviewers (N.L., V.C.S.). Discrepant findings were reassessed by consensus. Outcome measures were compared between the two cohorts to assess the feasibility, safety, and effectiveness of the standardized bundle.

Demographic and sedation data were entered manually or scanned into the electronic medical record (EMR). All data analyzed in this study were obtained from the EMR. Statistical analysis was performed using SPSS version 29.0 (IBM, SPSS Statistics, IBM Corporation, Chicago, IL, USA). Demographic and clinical data (e.g., age, weight) were reported as mean ± SD [range]. Descriptive data were reported as absolute numbers and proportions. Associations were analyzed using the chi‐squared test and Mann–Whitney *U*‐test. A *p*‐value ≤ 0.05 was considered statistically significant.

## 3 Results

Between December 2012 and June 2025, a total of 20 neonates underwent endoscopic PEBP adjustments. After excluding incomplete data sets, 18 cases were included in the final analysis, encompassing cardiopulmonary parameters, pharmacological analgosedation regimens, and perioperative complications. Eight infants (44.4%) were treated in Cohort I, managed with individualized analgosedation protocols. Ten infants (55.6%) were treated in Cohort II under the standardized analgosedation and procedural care bundle introduced in 2022.

All infants were born between 25 + 3 and 40 + 6 weeks of gestation, with a mean (± SD) birth weight of 3.036 ± 0.88 kg [range: 0.740–4.290 kg]. At the time of PEBP placement, the mean (± SD) body weight was 3.350 ± 0.68 kg [range: 2.250–4.620 kg]. The timing of the PEBP adjustment varied depending on referral patterns, clinical presentation, and comorbid conditions. Detailed patient characteristics, including pre‐existing conditions that contributed to delayed interventions, are summarized in Table 1.

**TABLE 1:** Patients' characteristics.

Case	ASA	Gender (M/F)	Gestational age at birth (weeks + days)	Body weight at birth (kg)	Post menstrual age at PEBP (weeks + days)	Body weight at PEBP (kg)	Medical special feature
Cohort I							
1	3	M	39 + 2	2.820	41 + 1	2.800	
2	2	F	39 + 0	3.810	40 + 5	3.300	
3	2	F	39 + 6	2.100	44 + 2	2.400	Postnatal palatal plate; interval presentation for PEBP evaluation
4	2	F	40 + 0	3.570	42 + 3	3.100	Secondary deterioration promting referral for PEBP
5	3	M	38 + 5	4.040	39 + 4	3.900	
6	3	M	39 + 6	3.000	50 + 0	4.020	Ductus arteriosus ligation and PICU stay prior to PEBP
7	3	F	38 + 0	2.400	40 + 3	2.250	
8	2	M	37 + 6	4.290	39 + 1	4.170	
Cohort II							
9	3	F	38 + 6	4.020	44 + 3	4.453	palatal plate; interval presentation for PEBP evaluation
10	2	F	40 + 6	3.510	42 + 0	3.500	
11	2	F	38 + 1	2.820	40 + 0	2.900	
12	3	M	40 + 3	3.560	43 + 6	3.600	Secondary deterioration prompting referral for PEBP
13	2	F	40 + 3	3.244	40 + 6	3.040	
14	2	M	29 + 5	1.865	44 + 2	3.500	Secondary transfer following neonatal monitoring due to prematurity
15	3	M	40 + 0	3.270	41 + 6	3.300	
16	2	F	38 + 2	2.695	39 + 3	2.610	
17	2	M	25 + 3	0.740	29 + 4	2.850	
18	2	F	36 + 4	2.890	49 + 3	4.620	External admission after non‐responsive alternative management

Abbreviations: ASA, American Society of Anesthesiologists physical status classification system; PEBP, pre‐epiglottic baton plate.

Table 2 presents a comparative overview of both cohorts. No statistically significant differences were observed between the groups in terms of disease severity, chronological age, or body weight at the time of intervention. Opioids were not included in the standardized analgosedation protocol, which explains group differences. Details of medications used for initiation and maintenance of analgosedation are summarized in Table 3. In Cohort I, drugs were selected on a case‐by‐case basis. In contrast, Cohort II followed a predefined analgosedation protocol consisting of sevoflurane for inhalational induction and maintenance with ketamine (1–2 mg/kg/h) and dexmedetomidine (1–2 μg/kg/h). Sevoflurane was also used intermittently during procedural pauses when additional sedation was required.

**TABLE 2:** Cohort characteristics and anesthesia procedure.

	Cohort I	Cohort II	*p*
*n* (%)	8 (44.4)	10 (55.6)	
Femal gender, *n* (%)	4 (50.0)	6 (60.0)	0.67
ASA, *n* (%)			0.39
ASA II	4 (50.0)	7 (70.0)	
ASA III	4 (50.0)	3 (30.0)	
Gestational age at birth, days, mean ± SD [range]	273.5 ± 5.9 [265–280]	258.1 ± 36.2 [178–286]	0.90
Post menstrual age at PEBP, days, mean ± SD [range]	295.5 ± 24.8 [274–350]	291 ± 35.7 [207–346]	0.76
Body weight at birth, kg, mean ± SD [range]	3.254 ± 0.79 [2.100–4.290]	2.861 ± 0.95 [0.740–4.020]	0.36
Body weight at PEBP, kg, mean ± SD [range]	3.240 ± 0.74 [2.250–4.170]	3.437 ± 0.66 [2.610–4.620]	0.57
Volatile anesthetics used during intervention, *n* (%)	3 (37.5)	10 (100.0)	0.03
Opioid used during intervention, *n* (%)	5 (62.5)	0 (0)	0.03
Remifentanil, *n* (%)	4 (50.0)	0 (0)	
Piritramid, *n* (%)	1 (12.5)	0 (0)	
Induction, minutes, mean ± SD [range]	8.8 ± 5.0 [2–15]	5.6 ± 2.7 [3–10]	0.26
Intervention, minutes, mean ± SD [range]	84.7 ± 34.3 [50–136]	38 ± 8.5 [26–55]	≤ 0.001

Abbreviation: ASA, American Society of Anesthesiologists physical status classification system.

**TABLE 3:** Procedural sedation and oxygen saturation.

No	Cohort	Place of intervention	Induction (drugs)	Maintenance (drugs)	SpO_2_ Air, %	SpO_2_ intra‐op lowest, %	SpO_2_ post‐op, %
1	I	POT	Sevoflurane	Propofol, remifentanil, dexmedetomedine	91	99	90
2	I	PICU	Propofol	Propofol	100	100	99
3	I	POT	Sevoflurane, propofol	Propofol, remifentanyl	99	95	96
4	I	POT	Propofol, remifentanyl	Propofol, remifentanyl	95	96	95
5	I	PICU	Midazolam, ketamine	Midazolam, ketamine	96	91	n.n.
6	I	POT	Propofol, remifantanyl	Propofol, remifantanyl	97	80	95
7	I	GOT	Midazolam	Midazolam, piritramide	100	98	98
8	I	PICU	Chloralhydrat	Ketamine	98	96	95
9	II	POT	Sevofluran	Ketamine, dexmedetomedine	98	98	99
10	II	POT	Sevefluran	Ketamine, dexmedetomedine	92	97	97
11	II	POT	Sevoflurane	Ketamine, dexmedetomedine	100	83	97
12	II	POT	Sevoflurane	Ketamine, dexmedetomedine	100	100	100
13	II	POT	Sevoflurane	Ketamine, dexmedetomedine	97	97	96
14	II	POT	Sevoflurane	Ketamine, dexmedetomedine	96	100	96
15	II	POT	Sevoflurane	Ketamine, dexmedetomedine	95	93	96
16	II	POT	Sevoflurane	Ketamine, dexmedetomedine	92	100	97
17	II	POT	Sevoflurane	Ketamine, dexmedetomedine	93	98	98
18	II	POT	Sevoflurane	Ketamine, dexmedetomedine	96	65	97

Abbreviations: GOT, general operating theater; PICU, pediatric intensive care unit; POT, pediatric operating theater.

Baseline peripheral oxygen saturation prior to analgosedation showed no significant difference between groups (*p* = 0.42), nor did the lowest intraoperative oxygen saturation (*p* = 0.63) and the first recorded saturation post‐intervention (*p* = 0.08). The mean (± SD) duration of analgesia induction was 8.83 ± 5.04 min [range: 2–15 min] in Cohort I and 5.60 ± 2.72 min [range: 3–10 min] in Cohort II, with no significant difference (*p* = 0.26). However, procedure duration was significantly shorter in Cohort II (*p* < 0.001): 84.67 ± 34.31 min [range: 50–136 min] in Cohort I versus 38 ± 8.54 min [range: 26–55 min] in Cohort II.

In Cohort I, 4 out of 8 procedures (50%) were performed in the specialized pediatric operating theater; the remainder occurred in the PICU or, in one case, a general OR. In contrast, all procedures in Cohort II were consistently performed in the designated pediatric operation theater setting, in accordance with the standardized bundle. Figure 2 illustrates the defined spatial layout and setup used.

**FIGURE 2:**
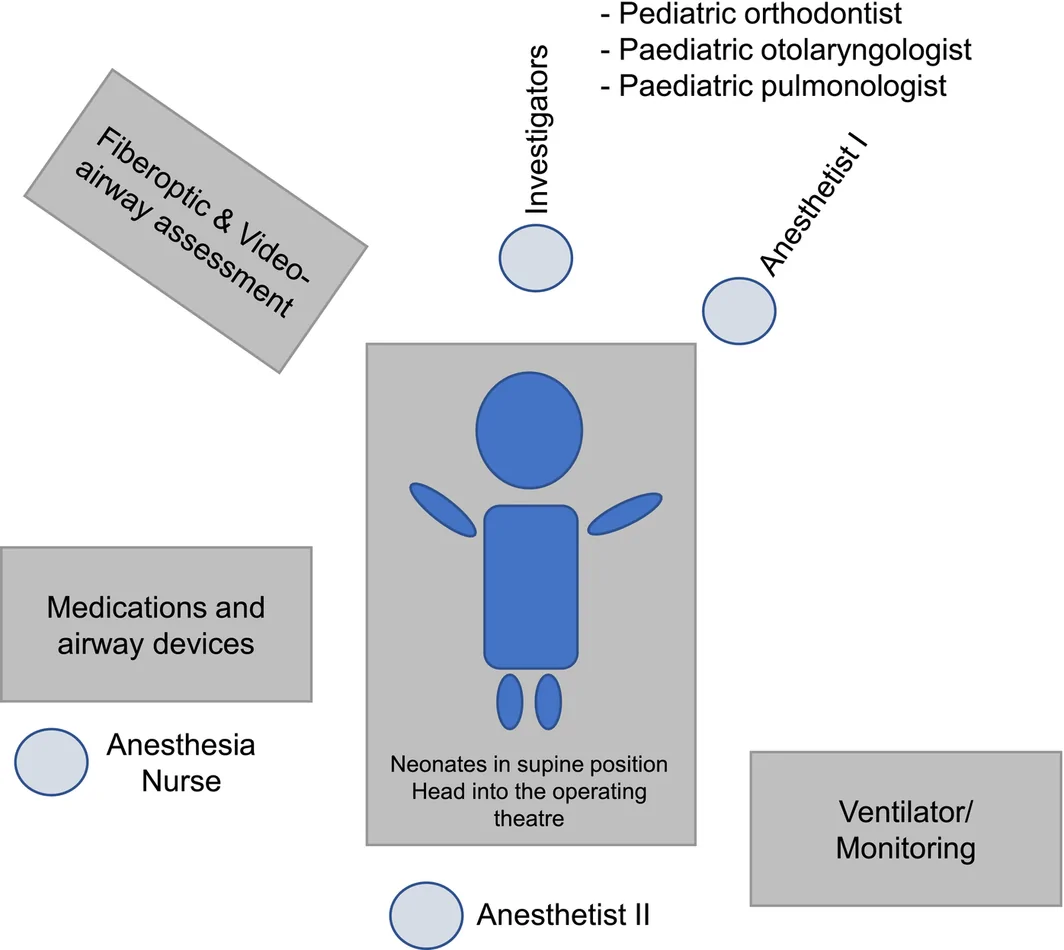
Operating theater setup. Figure 2 illustrates the operating theater setup featuring optimized positioning of medical equipment and investigators.

A total of 5 adverse events were documented, 4 in Cohort I and one in Cohort II. All were managed safely with basic maneuvers (jaw thrust, intermittent mask ventilation), without requiring emergency airway intervention (Table 4).

**TABLE 4:** Adverse events.

No	Cohort	Adverse event	Intervention/Impact
1	I	Inadequate spontaneous respiration	Intermittent mask ventilation, jaw thrust
2	I	Variable sedation depth	Jaw thrust after bolus doses
3	I	Inadequate spontaneous respiration	Intermittent mask ventilation, jaw thrust
4	I	Insufficient sedation (agitation)	Procedure delayed, supportive care
5	II	Laryngospasm with desaturation	Sedation deepened, intermittent mask ventilation

## 4 Discussion

Our findings in the present single‐center retrospective cohort analysis in a very small patient population show safe and effective analgosedation for endoscopic PEBP adjustment through a range of pharmacological strategies, even in this high‐risk neonatal population. The variability observed in analgosedation regimens prior to protocol implementation reflects both the rarity of the condition and the procedural challenges inherent to RS management. While tailored approaches may remain necessary in selected cases, the absence of standardization impaired reproducibility, team coordination, and procedural efficiency. In contrast, the implementation of a predefined multimodal analgosedation protocol, using low to moderate doses of sevoflurane, ketamine, and dexmedetomidine, was associated with improved procedural consistency and could potentially reduce the incidence of analgosedation‐related complications. These observations align with existing literature suggesting that combining sedative agents at moderate doses may offer greater safety margins than reliance on higher doses of single agents [20].

The lower number of adverse events in Cohort II may, in part, reflect the shorter intervention duration. At the same time, effective sedation and a well‐coordinated team within a standardized setting likely contributed to this reduction. Beyond pharmacological standardization, structural and organizational elements played a critical role in enhancing procedural safety by creating a consistent procedural environment and facilitating effective interdisciplinary coordination. The use of a designated pediatric operating theater with a defined spatial layout and optimized equipment setup improved access to the airway and enabled seamless collaboration among team members. A standardized team composition, including dual anesthetic staffing supported by experienced pediatric specialists, ensured continuity of expertise and allowed for timely responses to dynamic airway challenges. Additionally, perioperative care was consistently provided in the pediatric intensive care unit, with continuous cardiorespiratory monitoring and immediate availability of airway support. Together, these structural and organizational measures contributed to shorter procedure times and likely mitigated perioperative risks, underscoring the importance of a comprehensive, systematized approach to the management of neonates with Robin sequence undergoing PEBP adjustment.

Another important consideration is whether analgosedation is required for this procedure at all. Notably, other centers in Germany offering this treatment perform PEBP adjustment explicitly without analgosedation [14, 21, 22, 23]. Current evidence suggests that both awake endoscopy and endoscopy under analgosedation are valid approaches for evaluating upper airway obstruction in infants with Robin sequence [24]. The European guideline considers both methods acceptable, while noting that endoscopy under analgosedation may be more sensitive in identifying glossoptosis but also introduces anesthesia‐related risks [25]. Lee et al. directly compared awake endoscopy and endoscopy under analgosedation, highlighting this central trade‐off between potentially greater diagnostic yield under sedation and the risk of airway compromise in an already vulnerable population [24]. In our center, however, the accumulated experience of the interdisciplinary team has favored a sedated approach, as it has been considered more feasible and safer in this particularly challenging clinical setting. This study has several limitations. Its retrospective design limits the level of evidence and carries a risk of incomplete documentation, particularly regarding perioperative details. The small sample size reflects the rarity of RS and the specialized nature of the intervention, reducing statistical power and generalizability. The extended study period may have introduced bias due to evolving clinical practices and team composition. Additionally, variability in analgosedation regimens prior to standardization could have influenced outcomes in ways that were not fully controllable.

Furthermore, the transferability of this protocol to other institutions may be limited by the extensive senior staffing requirements for an infrequently performed procedure.

Prospective studies with larger cohorts are needed to confirm these findings and refine analgosedation strategies for endoscopic airway procedures in neonates with RS.

In conclusion, endoscopic adjustment of the PEBP in neonates with RS can be performed under procedural analgosedation when embedded in a structured, interdisciplinary care framework. A standardized analgosedation protocol, defined team composition, and consistent procedural setting may contribute to greater procedural consistency and could help reduce the incidence of adverse events. This approach appears to support a high level of procedural efficiency while maintaining a clear focus on patient safety. Prospective studies are warranted to validate these findings and refine evidence‐based care pathways for this high‐risk population.

## Author Contributions

N.L. and V.C.S. contributed equally to the design of study, data acquisition, data analysis, manuscript drafting, and revision. S.A., U.T., T.K., and B.B. contributed to the study design and critically revised the manuscript. All authors (N.L., V.C.S., S.A., U.T., T.K., B.B.) reviewed and approved the final version of the manuscript.

## Funding

The authors have nothing to report.

## Ethics Statement

This retrospective cohort study was approved by the ethics committee of the University Hospital of Cologne, Cologne, Germany (Reference No. 23–1145, Amendment 2025).

## Consent

The requirement for informed consent was waived by the committee due to the retrospective nature of the study.

## Conflicts of Interest

The authors declare no conflicts of interest.

## Data Availability

The data that support the findings of this study are available from the corresponding author upon reasonable request.
